# Antibody Maturation and Viral Diversification in HIV-Infected Women

**DOI:** 10.1371/journal.pone.0057350

**Published:** 2013-02-27

**Authors:** Maria M. James, Oliver Laeyendecker, Jin Sun, Donald R. Hoover, Caroline E. Mullis, Matthew M. Cousins, Thomas Coates, Richard D. Moore, Gabor D. Kelen, Mary Glenn Fowler, Johnstone J. Kumwenda, Lynne M. Mofenson, Newton I. Kumwenda, Taha E. Taha, Susan H. Eshleman

**Affiliations:** 1 Department of Pathology, Johns Hopkins Univ. School of Medicine, Baltimore, Maryland, United States of America; 2 Laboratory of Immunoregulation, National Institute of Allergy and Infectious Diseases, National Institutes of Health, Bethesda, Maryland, United States of America; 3 Dept. of Medicine, Johns Hopkins University School of Medicine, Baltimore, Maryland, United States of America; 4 Dept. of Epidemiology, Johns Hopkins University Bloomberg School of Public Health, Baltimore, Maryland, United States of America; 5 Department of Statistics and Biostatistics and Institute for Health, Health Care Policy and Aging Research, Rutgers University, Piscataway, New Jersey, United States of America; 6 Program in Global Health, University of California Los Angeles, Los Angeles, California, United States of America; 7 Dept. of Emergency Medicine, Johns Hopkins Univ. School of Medicine, Baltimore, Maryland, United States of America; 8 Dept. of Medicine, College of Medicine, University of Malawi, Blantyre, Malawi; 9 Pediatric, Adolescent, and Maternal AIDS Branch, Eunice Kennedy Shriver National Institute of Child Health and Human Development, National Institutes of Health, Rockville, Maryland, United States of America; University of Amsterdam, The Netherlands

## Abstract

**Introduction:**

The Post-exposure Prophylaxis in Infants (PEPI)-Malawi trial evaluated infant antiretroviral regimens for prevention of post-natal HIV transmission. A multi-assay algorithm (MAA) that includes the BED capture immunoassay, an avidity assay, CD4 cell count, and viral load was used to identify women who were vs. were not recently infected at the time of enrollment (MAA recent, N = 73; MAA non-recent, N = 2,488); a subset of the women in the MAA non-recent group known to have been HIV infected for at least 2 years before enrollment (known non-recent, N = 54). Antibody maturation and viral diversification were examined in these women.

**Methods:**

Samples collected at enrollment (N = 2,561) and 12–24 months later (N = 1,306) were available for serologic analysis using the BED and avidity assays. A subset of those samples was used for analysis of viral diversity, which was performed using a high resolution melting (HRM) diversity assay. Viral diversity analysis was performed using all available samples from women in the MAA recent group (61 enrollment samples, 38 follow-up samples) and the known non-recent group (43 enrollment samples, 22 follow-up samples). Diversity data from PEPI-Malawi were also compared to similar data from 169 adults in the United States (US) with known recent infection (N = 102) and known non-recent infection (N = 67).

**Results:**

In PEPI-Malawi, results from the BED and avidity assays increased over time in the MAA recent group, but did not change significantly in the MAA non-recent group. At enrollment, HIV diversity was lower in the MAA recent group than in the known non-recent group. HRM diversity assay results from women in PEPI-Malawi were similar to those from adults in the US with known duration of HIV infection.

**Conclusions:**

Antibody maturation and HIV diversification patterns in African women provide additional support for use of the MAA to identify populations with recent HIV infection.

## Introduction

Serologic assays have been developed to estimate HIV incidence, based on the premise that the anti-HIV antibody response matures over time in infected individuals [1], [2]. The BED capture immunoassay measures the proportion of IgG that is HIV specific [3], and an avidity assay based on the Genetic Systems HIV-1/HIV-2+ O EIA assay measures the strength with which anti-HIV antibodies bind to target antigens [4], [5]. These and other serologic incidence assays have been shown to misclassify some individuals with non-recent infection as recently infected [6], [7], [8]. We developed a multi-assay algorithm (MAA) for HIV incidence estimation that combines these two serologic assays with CD4 cell count and HIV viral load [9]. In the United States (US), where HIV-1 subtype B is prevalent, this MAA has a very low rate of false-recent misclassification and provides cross-sectional HIV incidence estimates that are very similar to those obtained from longitudinal follow-up of study cohorts [9], [10]. We used this MAA to analyze samples from the Post-exposure Prophylaxis of Infants (PEPI)-Malawi trial [11], which enrolled over 3,000 HIV-infected pregnant women who were followed for 18–24 months [12]. Women in this trial were likely to have been infected with HIV-1 subtype C [13], [14]. The MAA identified 73 women as recently infected at the time of enrollment (MAA recent group); those women had a significantly higher rate of *in utero* HIV transmission than did women who were identified as having non-recent HIV infection at enrollment (MAA non-recent group) [12]. This finding supported use of the MAA for identification of individuals with recent HIV infection. Demographic data from PEPI-Malawi also supported use of the MAA for identifying recently-infected individuals; women identified as recently infected using the MAA were younger and had lower parity than women identified as not recently infected [12].

The level of HIV diversity may also be useful for identifying individuals with recent HIV infection [15], [16], [17]. We developed an assay based on high resolution melting (HRM) that quantifies the HIV diversity without sequencing [18], [19]. The HRM diversity assay has been optimized for analysis of multiple regions of the HIV genome [15], [19], [20]. Results obtained using the HRM diversity assay are highly correlated with those obtained using next generation sequencing [21]. In a previous study, we used the HRM diversity assay to compare diversity in six regions of the HIV genome among adults from the US with known recent and known non-recent HIV infection [15]; these adults were likely to have HIV-1 subtype B infection. Viral diversity was significantly lower in the known recent group in five of the six regions analyzed [15].

In this study, we used the BED assay, the avidity assay, and the HRM diversity assay to compare antibody maturation and viral diversity in women in the PEPI-Malawi cohort. This cohort included women who were identified as recently infected or not recently infected using the MAA, as well as women with known non-recent HIV infection. This is the largest study to date evaluating longitudinal changes in BED and avidity test results in adults classified as having recent vs. non-recent infection using the MAA.

## Methods

### Ethics Statement

Written informed consent was obtained from all women enrolled in the PEPI-Malawi trial (conducted in 2004–2009, NCT00115648). The PEPI-Malawi trial was approved by Institutional Review Boards in Malawi and the US, as previously described [11].

### Study Population and Source of Samples

**Figure 1:**
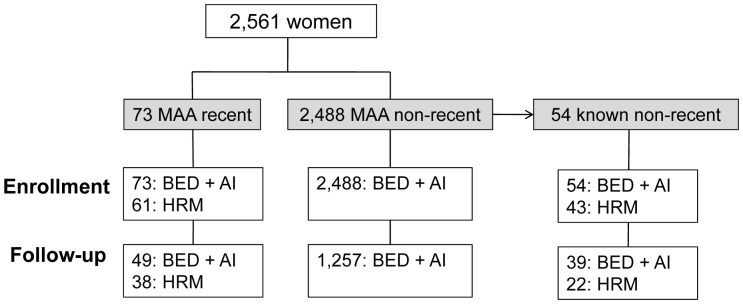
Samples used for analysis (see text). Samples collected at study enrollment were available for 2,561 (76.8%) of the 3,335 women enrolled in the PEPI-Malawi trial. The figure shows the number of samples available for analysis using the BED assay (BED), the avidity assay (avidity index, AI) and the HRM diversity assay (HRM) at enrollment and follow-up (12–24 months after enrollment). Different subsets of women were evaluated, including women identified as recently infected at enrollment using the multi-assay algorithm (MAA recent), women identified as not recently infected at enrollment using the MAA (MAA non-recent), and women who were known to have non-recent HIV infection at enrollment (known non-recent).

Plasma samples were obtained from HIV-infected women enrolled in the PEPI-Malawi trial (enrollment: 2004–2009) [11]. Samples collected at study enrollment were available for 2,561 (76.8%) of the 3,335 women (Figure 1). In a previous study, 73 of the 2,561 women were classified as recently infected at enrollment using the MAA (MAA recent) [12]. Recent infection was defined by having all of the following test results: BED <1.0 normalized optical density units (OD-n), avidity index <80%, CD4 cell count >200 cells/mm^3^, and HIV viral load >400 copies/mL. This MAA has a positive predictive value of 93.9% for identifying recent HIV infections in populations where HIV-1 subtype C is prevalent [12]. Recent studies indicate that the mean duration of the recent period (window period) for this MAA is 6–7 months in populations with HIV-1 subtype A and C infection. The remaining 2,488 women were classified as not recently infected using the MAA (MAA non-recent). Fifty-four of those 2,488 women had been diagnosed with HIV infection 2–6 years earlier in a previous clinical trial [12] (known non-recent). This study analyzed samples collected at enrollment and 12–24 months after enrollment. Follow-up samples collected 12–24 months after enrollment were available for 1,306 (51%) of the 2,561 women (one follow-up sample for each woman: 21 samples from the 12-month visit, one sample from the 15-month visit, 67 samples from the 18-month visit, and 1,217 samples from the 24-month visit). The majority of women in the PEPI-Malawi trial (∼70%) received single dose nevirapine in labor. None were receiving antiretroviral treatment at enrollment, and only nine (0.35%) of the 2,561 women initiated antiretroviral treatment during the trial; these nine women (all in the MAA non-recent group) received a regimen of stavudine, lamivudine and nevirapine. Women in the PEPI-Malawi trial were likely to have been infected with HIV-1 subtype C [13], [14]. In a previous study of pregnant HIV-infected women enrolled at the same study site, all of the infections analyzed were HIV-1 subtype C [22].

The HRM diversity assay was also used to analyze samples collected from adults in the United States. These samples were collected from the HPTN 015 study (the EXPLORE Study), the Johns Hopkins HIV Clinical Cohort study, and the Johns Hopkins Hospital Emergency Department HIV serosurvey [15]. These adults are likely to have been infected with HIV-1 subtype B [23]. In a previous study, 102 (60%) of the 169 individuals from the US described in this report were confirmed to have HIV-1 subtype B infection [24].

### Analysis of the Serologic Response to HIV Infection

The BED assay was performed according to the manufacturer’s instructions with one exception, samples were run in duplicate and results were reported as mean normalized optical density units (Sedia Biosciences Corporation, Portland, OR, USA). The avidity assay, a modified version of the Genetic Systems HIV-1/HIV-2+ O EIA (Bio-Rad Laboratories, Redmond, WA), was performed as previously described [12].

### Analysis of HIV Diversity

The HRM diversity assay was performed as previously described [18], [19]. Briefly, DNA from the *gag-pol* and *env* regions was amplified. The amplicons were used as templates for a nested polymerase chain reaction (PCR); the nested PCR step included a fluorescent dye that was incorporated into the amplified products. The samples were analyzed using a LightScanner instrument (Model HR 96, BioFire Diagnostics, Inc., Salt Lake City, UT), which heats the samples and measures the change in fluorescence associated with DNA duplex melting. The range of temperatures over which melting occurs is defined as the HRM score [18], [19]. Six genomic regions were analyzed: GAG1, GAG2, POL, ENV1, ENV2, and ENV3 [15].

### Statistical Analysis

The outcome variables of interest were not normally distributed; therefore, rank tests were used for statistical comparison. BED OD-n values, avidity index values, and HRM scores at enrollment and at follow-up were compared between MAA recent and non-recent infection groups using Wilcoxon rank sums. The Wilcoxon rank sum test was also used to compare the time between the enrollment and follow-up visits in different subgroups of women. Paired analyses of enrollment and follow-up data from each group were performed using signed rank tests. Statistical analyses were done using SAS software version 9.3 (SAS institute, Cary, NC, USA).

## Results

### Samples used for Analysis

We analyzed samples collected at enrollment from 2,561 women in the PEPI-Malawi trial, including 73 women whom were previously identified as recently infected at enrollment using the MAA (MAA recent) and 2,488 women who were identified as not recently infected (MAA non-recent) [12] (Figure 1). Fifty-four women in the MAA non-recent group had been diagnosed with HIV infection at least two years prior to enrollment in the PEPI-Malawi trial and were classified as known non-recent. Follow-up samples, collected 12–24 months after study enrollment, were available for 1,306 (51%) of the 2,561 women: 49 in the MAA recent group and 1,257 in the MAA non-recent group (Figure 1). The number of days between the enrollment and follow-up visits was similar for these two groups (median [IQR] of 730 [719, 731] for MAA recent and 730 [729, 732] for MAA non-recent, P = 0.08). All available enrollment and follow-up samples were analyzed using the BED and avidity assays. HRM diversity analysis was performed for women in the MAA recent and known non-recent groups; 104 enrollment samples and 60 follow-up samples were available for HRM diversity analysis (Figure 1). The number of days between the enrollment and follow-up visits for women in the MAA recent group versus known non-recent group who were included in the HRM diversity analysis was similar (median [IQR] of 729 [366,731] for MAA recent and 729 [365,732] for MAA non-recent, P = 0.98).

### Serologic Assays

The serologic response to HIV infection was evaluated using the BED and avidity assays; these assays are part of the MAA used to identify women with recent HIV infection. As defined by the MAA, all women in the MAA recent group had BED <1.0 OD-n and avidity index <80% at the time of enrollment, while women in the MAA non-recent group generally had higher BED and/or avidity values (Table 1). At follow-up (12–24 months after study enrollment), BED and avidity test results were still statistically lower in the MAA recent group than the MAA non-recent group (P<0.0001 for both assays, Table 1). Qualitative differences were noted in the results obtained using these two assays. At the follow-up visit, the median avidity values in the MAA recent and MAA non-recent groups were qualitatively similar (98.60% vs. 99.58%). The low p value (<0.0001) for this comparison reflects the difference in the lower quartile avidity values for the MAA recent group (82.37%) compared to the MAA non-recent group (95.58%). In contrast, the median BED value in the MAA recent group was still much lower at follow-up than was the median BED value in the MAA non-recent group (1.10 vs. 2.51 OD-n). These qualitative differences suggest that avidity test results increase more quickly and in a higher proportion of recently infected women than BED test results.

**Table 1:** Comparison of BED and avidity test results at enrollment and follow-up in women with MAA recent versus MAA non-recent HIV infection.*

	MAA Recent		MAA Non-recent		P value^b^
	N	Median (IQR^a^)	N	Median (IQR^a^)	
BED at enrollment	73	0.55 (0.40, 0.76)	2,488	2.63 (1.74, 3.52)	<0.0001
BED at follow-up	49	1.10 (0.68, 1.70)	1,257	2.51 (1.61, 3.43)	<0.0001
Avidity at enrollment	73	35.39 (23.38, 62.43)	2,488	99.5 (98.33, 100.11)	<0.0001
Avidity at follow-up	49	98.60 (82.37, 99.66)	1,257	99.58% (95.58, 100.41)	<0.0001

* BED results are expressed as OD-n (normalized optical density) and avidity results are expressed as the avidity index (%).

a IQR: Interquartile range.

b P values were calculated using Wilcoxon rank sum test.

**Figure 2:**
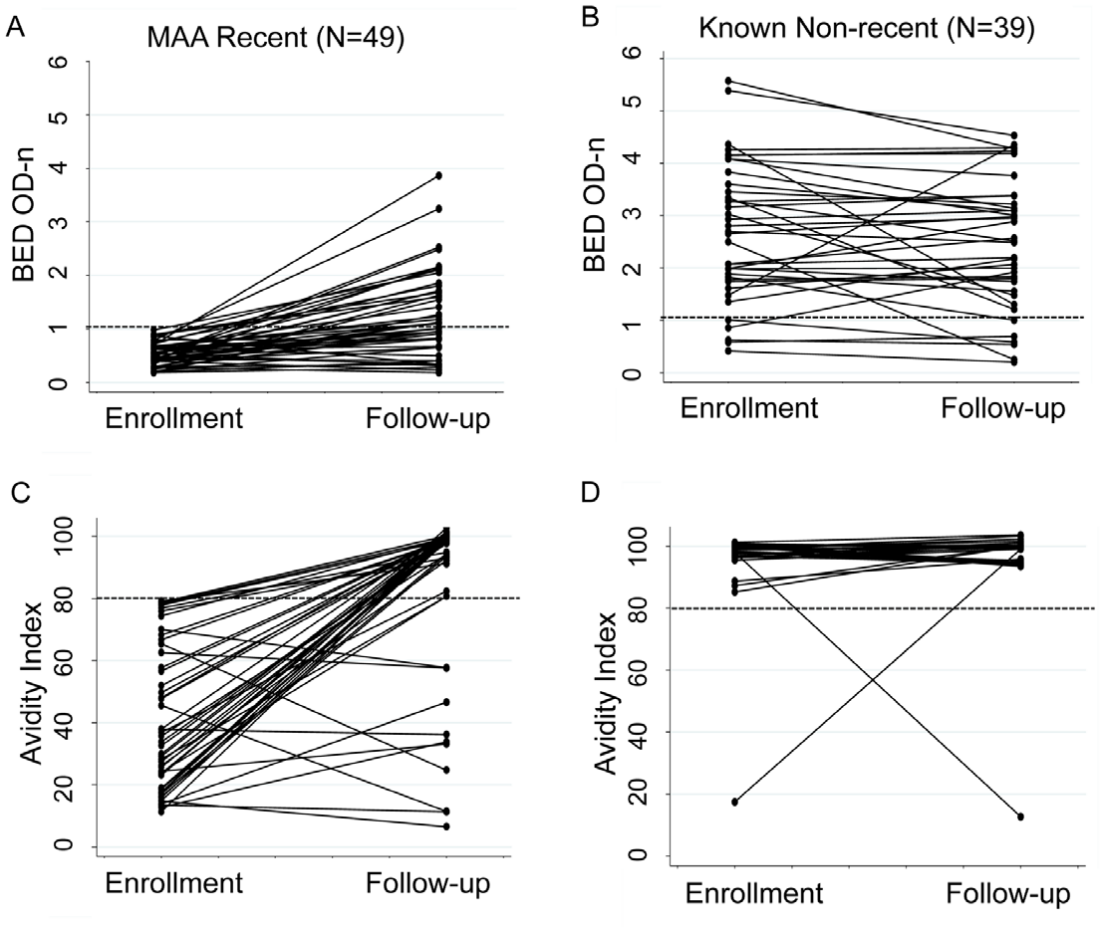
Comparison of BED and avidity test results obtained at enrollment and at study follow-up. The figure shows results obtained for the BED assay (Panels A and B) and the avidity assay (Panels C and D) at enrollment and at follow-up (12–24 months after enrollment) for two groups of women: women identified as recently infected at enrollment using the MAA (MAA recent, Panels A and C), and women who were known to have non-recent HIV infection at enrollment (known non-recent, Panels B and D). Data are shown only for women who had test results obtained at both study visits (paired data). Dotted lines indicate the cutoffs used for the BED and avidity assay in the MAA (1.0 OD-n and 80% avidity index, respectively).

We next compared changes in BED and avidity results from enrollment to follow-up (paired analysis, Table 2). In the MAA recent group (among 49 women who had follow-up samples), results from both the BED and avidity assays increased significantly over time (P<0.0001 for both assays; median BED increase: 0.56 OD-n; median avidity index increase: 50.74%, Table 2). In the MAA non-recent group (among 1,257 women who had follow-up samples), BED results decreased over time (P<0.0001), while avidity results increased over time (P = 0.003); however, the magnitude of those changes was small (median BED decrease: 0.11 OD-n; median avidity increase: 0.02%, Table 2, Figure 2).

**Table 2:** Analysis of antibody maturation in women with MAA recent versus MAA non-recent HIV infection (paired analysis).*

	MAA Recent (N = 49)		MAA Non-recent (N = 1,257)		Comparing changes
	Change from enrollment tofollow-up^a^	P value^b^	Change from enrollment tofollow-up^a^	P value^b^	P value^c^
BED	0.56 (0.19, 1.26)	<0.0001	−0.11 (−0.53, 0.29)	<0.0001	<0.0001
Avidity	50.74 (21.05, 74.28)	<0.0001	0.02 (−2.38, 1.47)	0.003	<0.0001

* BED results are expressed as OD-n (normalized optical density) and avidity results are expressed as the avidity index (%).

a Median paired difference of results at enrollment and follow-up; interquartile ranges are shown in parentheses.

b P values were calculated using Wilcoxon signed rank test.

c P values were calculated using Wilcoxon rank sum test.

Figure 2 provides a graphical display of BED and avidity results from 49 women in the MAA recent group who had follow-up samples, and from the subset of 39 women in the MAA non-recent group who had follow-up samples and were known to have been infected for at least two years at the time of enrollment in the PEPI-Malawi trial (known non-recent). This figure shows that most of the women in the MAA recent group had BED and avidity values that increased over time; 46 (93.8%) of the 49 women in the MAA recent group who had follow-up samples were classified by the MAA as non-recent at the follow-up visit. Three of the 49 women had results below the cut-off for the BED and avidity assays at follow-up. In contrast, most women in the known non-recent group had BED and avidity values that did not change significantly during follow-up.

### HIV Diversity Analysis

The HRM diversity assay was used to analyze *gag*, *pol* and *env* diversity in women in the MAA recent and known non-recent groups (Figure 1). The MAA used to classify women as recently infected does not include an HIV diversity assay; therefore, HRM scores provide an independent method for assessing recent HIV infection. At enrollment, HRM scores (reflecting the level of genetic diversity in the HIV populations) were significantly lower in the MAA recent group in all six regions analyzed (P<0.0001 for four regions, P = 0.007 for POL, P = 0.01 for ENV3, Table 3). In contrast, at follow-up visits, HRM scores were significantly lower in the MAA recent group for only three regions (POL: P = 0.003, ENV1: P = 0.03, and ENV2: P = 0.04, Table 4).

**Table 3:** Comparison of HIV diversity in women with MAA recent versus known non-recent HIV infection at enrollment.*

Region analyzed	MAA Recent (N = 38)	Known Non-recent (N = 22)	P value^b^
	Median (IQR^a^)	Median (IQR^a^)	
GAG1	4.46 (4.35, 4.73)	5.56 (5.32, 6.12)	<0.0001
GAG2	4.48 (4.29, 5.16)	6.39 (5.30, 8.20)	<0.0001
POL	4.44 (4.04, 4.81)	5.29 (4.47, 5.95)	0.007
ENV1	4.50 (4.38, 4.83)	5.58 (4.81, 6.39)	<0.0001
ENV2	4.46 (4.33, 4.79)	5.74 (5.32, 6.57)	<0.0001
ENV3	4.81 (4.49, 5.91)	5.89 (5.23, 6.54)	0.01

* HIV diversity was measured using a high resolution melting (HRM) diversity assay, which expresses the genetic diversity in each region analyzed as a single numeric HRM score. The median and IQR values for HRM scores are shown.

a IQR: Interquartile range.

b P values were calculated using Wilcoxon rank sum test.

**Table 4:** Comparison of HIV diversity in women with MAA recent versus known non-recent HIV infection at follow-up.*

	MAA Recent (N = 38)	Known Non-recent (N = 22)		
Region analyzed	Median (IQR^a^)	Median (IQR^a^)	P value^b^	
GAG1	4.90 (4.50, 5.30)	5.40 (4.50, 5.80)	0.10	
GAG2	5.20 (4.50, 5.60)	5.55 (5.10, 6.20)	0.08	
POL	4.70 (4.40, 5.20)	5.25 (5.00, 5.50)	0.003	
ENV1	4.45 (4.22, 4.91)	4.94 (4.43, 6.40)	0.03	
ENV2	4.92 (4.34, 5.62)	5.76 (4.70, 6.64)	0.04	
ENV3	5.17 (4.80, 5.93)	5.40 (4.61, 6.80)	0.76	

* HIV diversity was measured using a high resolution melting (HRM) diversity assay, which expresses the genetic diversity in each region analyzed as a single numeric HRM score. The median and IQR values for HRM scores are shown.

a IQR: Interquartile range.

b P values were calculated using Wilcoxon rank sum test.

Additional analyses were performed to evaluate changes in HIV diversity over time in these two groups of women (from baseline to 12–24 months, paired analysis, Table 5). In the MAA recent group, there were significant increases in HRM scores over time in four of the six regions analyzed (GAG1, GAG2, POL and ENV2). In contrast, in the known non-recent group, there were significant decreases in HRM scores over time in three of the six regions (GAG1, GAG2, and ENV1). The patterns of diversification (changes in HRM scores over time) were significantly different in women with MAA recent vs. known non-recent infection in four regions (GAG1, GAG2, ENV1, and ENV2, Table 5).

**Table 5:** Comparison of the change in HIV diversity between enrollment and follow-up in women in the MAA recent and known non-recent groups (paired analysis).

Region analyzed	MAA Recent (N = 38)		Known Non-recent (N = 22)		Comparing changesP value^c^
	Change from enrollmentto follow-up^a^	P value^b^	Change from enrollmentto follow-up^a^	P value^b^	
GAG1	0.26 (0.04, 0.63)	<0.0001	−0.53 (−1.13, 0.28)	0.04	0.0007
GAG2	0.34 (−0.23, 1.12)	0.002	−0.74 (−2.0, 0.19)	0.04	0.001
POL	0.37 (−0.02, 0.66)	0.0004	−0.08 (−0.36, 0.69)	0.93	0.13
ENV1	−0.05 (−0.29, 0.26)	0.87	−0.32 (−0.75, 0.01)	0.004	0.03
ENV2	0.28 (−0.38, 0.96)	0.04	−0.74 (−1.39, 0.88)	0.33	0.04
ENV3	0.33 (−0.36, 1.23)	0.29	−0.41 (−1.50, 1.84)	0.89	0.57

** HIV diversity was measured using a high resolution melting (HRM) diversity assay, which expresses the genetic diversity in each region analyzed as a single numeric HRM score.

a Median paired difference of HRM scores at enrollment and follow-up; interquartile ranges are shown in parentheses.

b P values were calculated using Wilcoxon sign rank test for within group change being zero.

c P values were calculated using Wilcoxon rank sum test for changes being the same for the MAA recent and known non-recent groups.

### Comparison of HIV Diversity Results from the PEPI-Malawi Trial with Results Obtained for Adults in the US with known Duration of HIV Infection

**Figure 3:**
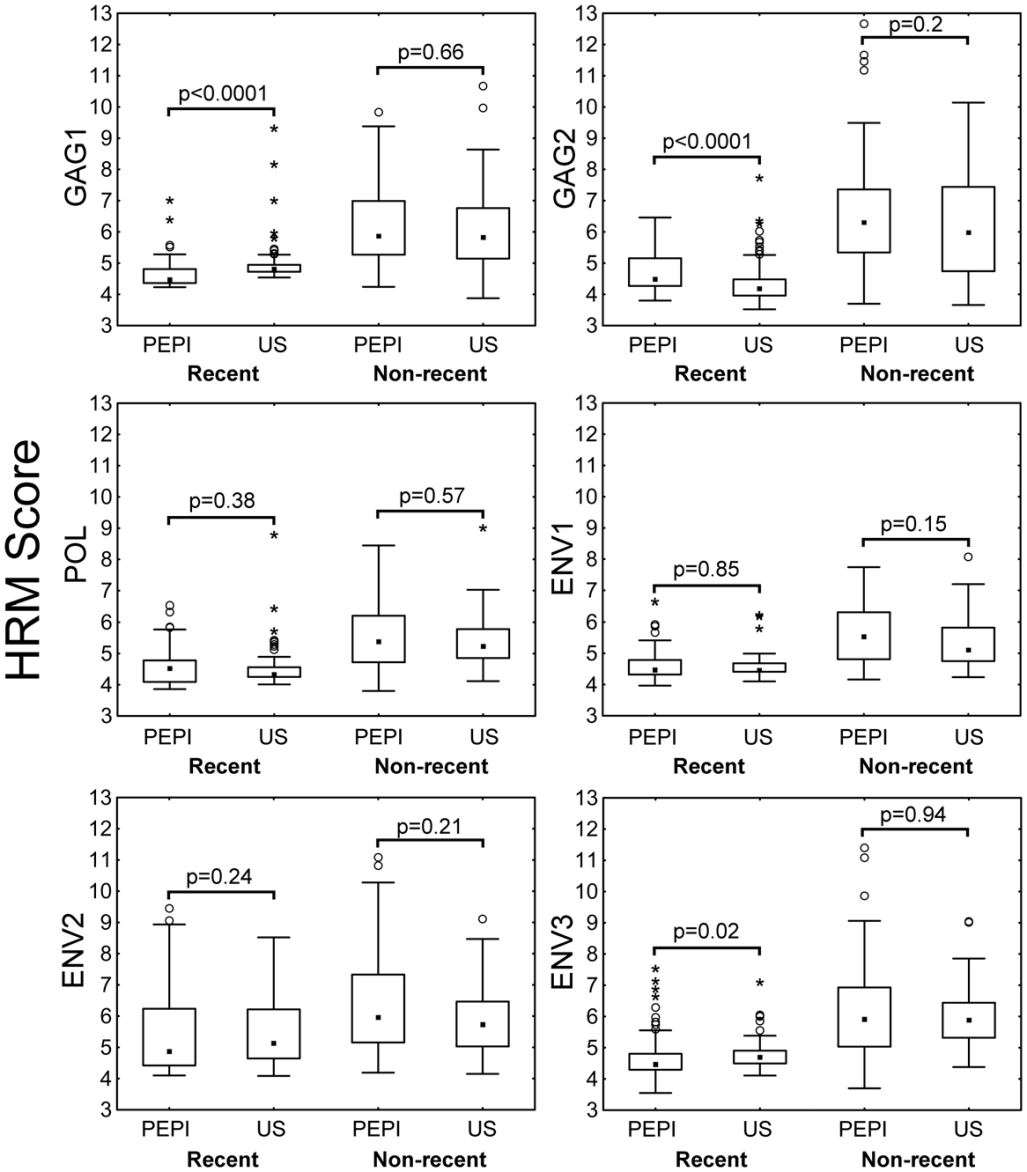
Comparison of HRM diversity in women in the PEPI-Malawi trial and adults in the United States. The figure compares HRM test results obtained for two groups: women in the PEPI-Malawi trial (PEPI, analyzed in this report) and adults in the United States (US, analyzed in a previous study [15]). Results from the PEPI-Malawi trial are shown for women identified as recently infected at enrollment using the MAA (MAA recent, N = 73), and women who were known to have non-recent HIV infection at enrollment (known non-recent, N = 54); these data were obtained by testing samples collected at the time of study enrollment. Results from adults in the US are shown for adults with known recent HIV infection (N = 102, samples collected at the time of HIV seroconversion, median time since last negative HIV test: 189 days, range 14–540 days) and adults with known non-recent HIV infection (N = 67, known to be infected for 2 years or more, including 32 with CD4 cell counts <50 cells/mm^3^ and 30 on antiretroviral therapy) [15]. P values were calculated using the Wilcoxon rank sum test.

In a previous report, we found significant differences in the pattern of HIV diversity among adults in the US with known recent vs. known non-recent HIV infection [15]. We compared HRM scores for women in the PEPI-Malawi trial to HRM scores for the adults in the US described in our previous report (Figure 3). The overall patterns of *gag*, *pol*, and *env* diversity were qualitatively similar in women from the PEPI-Malawi trial (pregnant women, likely to have been infected with HIV-1 subtype C; recent infection defined by the MAA) and adults from the US (men and women with different risk factors for HIV infection, likely to have been infected with HIV-1 subtype B; recent infection documented by HIV seroconversion). It should be noted that small, but statistically significant differences were observed for GAG1, GAG2, and ENV3 for MAA recent women in PEPI vs. known recent adults in the US, with a lower median score for the MAA recent women in PEPI in two of the three regions (GAG1, ENV3).

## Discussion

We previously used the MAA to identify 73 women in the PEPI-Malawi trial who were recently infected at the time of study enrollment (pregnant women, near the time of delivery) [12]. In analyses of other cohorts of adults with HIV-1 subtype C infection where the duration of HIV infection was known, this MAA had a positive predictive value of 93.9% for identifying recent infection [12]. Clinical data (a higher rate of *in utero* HIV transmission) and demographic data (younger age) also supported the classification of these 73 women as recently infected at the time of study enrollment [12]. Data from this report provides additional biological evidence supporting this classification.

The MAA includes two serologic assays: the BED assay and an avidity assay. All individuals classified as recently infected by the MAA have BED results <1.0 OD-n and avidity results <80%, while most individuals classified as not recently infected by the MAA have higher BED and/or avidity values. At enrollment, we observed the expected differences in BED and avidity values in the two groups, with lower values in the recent group (P<0.0001 for both assays). BED and avidity values increased significantly over the next 12–24 months in the recent group (P<0.0001 for both assays), while changes in serologic test results were negligible for the non-recent group. These differences in antibody maturation are similar to those observed in a smaller cohort of women in the United States who had documented HIV seroconversion (known recent) or were classified as recent or non-recent using the same MAA [10].

Analysis of HIV diversity and diversification over time, assessed using the HRM diversity assay, provided additional support for the MAA. HIV diversity is not part of the MAA and therefore serves as an independent biomarker for recent HIV infection. At enrollment, HIV diversity was significantly lower in the recent group in all six regions of the HIV genome; lower HIV diversity was still observed in this group 12–24 months later in three of the six regions (POL, ENV1, ENV2). Furthermore, women in the MAA recent group had an increase in viral diversity over 12–24 months (most notable for GAG1 and GAG2, also seen for POL and ENV2), while those in the known non-recent group did not. Taken together, these findings provide additional support for use of the MAA to identify individuals with recent HIV infection.

The levels of HIV diversity (HRM scores in each region) and pattern of HIV diversity (HRM scores in different genomic regions) that we observed in the MAA recent group (women classified as recently infected by the MAA) and known non-recent group (women known to have been infected for at least 2 years before study enrollment) were qualitatively similar to results obtained from adults in the US with known recent and non-recent HIV infection [15]. A previous study suggested that there may be gender-based differences in HIV diversity in early HIV infection [25], [26]. In this study, we did not find any meaningful differences in HRM scores for women in the PEPI-Malawi study (MAA recent group) vs. men who have sex with men (known recent group in the US cohort). The current study extends the previous study by including a larger number of women and men with recent HIV infection (73 women and 102 men) and by analyzing HIV diversity in *env*, *gag*, and *pol*. In this study, the similarity of results from the PEPI-Malawi trial (individuals likely to be infected with HIV-1 subtype C) and the US cohorts (individuals likely to be infected with HIV-1 subtype B) also suggest that HIV subtype has relatively little impact on the HIV diversity. However, we recognize that differences in diversity due to gender, HIV subtype, or other factors (e.g., risk factors for HIV acquisition, race) may not have been detected in this study because the two cohorts (PEPI and US) differed by several factors, which could have cancelled each other out. Further analysis is needed to evaluate the possible association of these factors with viral diversity.

The MAA used in this report was initially developed for HIV incidence estimation in populations with HIV-1 subtype B infection [9]. In previous studies of other cohorts, HIV incidence estimates obtained by longitudinal analysis of HIV seroconversion were very similar to those obtained by cross-sectional analysis using this MAA [9], [10], [27]. A similar MAA was used for cross-sectional analysis of HIV incidence in African populations in the HPTN 043 trial (Project Accept) [28]. Studies are underway to optimize a MAA for cross-sectional HIV incidence determination in populations with predominantly HIV-1 subtype C infection. Studies are also underway to evaluate incorporation of HIV diversity (measured using the HRM diversity assay) into MAAs for HIV incidence estimation, which might simplify those algorithms by eliminating the need for CD4 cell count data [15].
